# A Facile and Efficient Procedure for the Synthesis of New Benzimidazole-2-thione Derivatives

**DOI:** 10.3390/molecules17078578

**Published:** 2012-07-17

**Authors:** Augusto Rivera, Mauricio Maldonado, Jaime Ríos-Motta

**Affiliations:** Departamento de Química, Facultad de Ciencias, Universidad Nacional de Colombia, Sede Bogotá, Cra.30 No.45-03, Bogotá 111321, Colombia; Email: mmaldonadov@unal.edu.co (M.M.); jariosmo@unal.edu.co (J.R.-M.)

**Keywords:** benzimidazole-2-thione, benzimidazole, sulfur chemistry, aminal cage

## Abstract

A series of benzimidazole-2-thione derivatives was synthesized using a reaction between the macrocyclic aminal 16*H*,13*H*-5:12,7:14-dimethanedibenzo[*d,i*]-[1,3,6,8] tetraazecine (DMDBTA, **5**) and various nucleophiles in the presence of carbon disulfide. A full chemical characterization using IR, ^1^H-, ^13^C-NMR and GC-MS analyses of the new compounds is provided. These compounds were separated from the reaction mixture by column chromatography (CC) in highly pure form in 15%–51.4% yield.

## 1. Introduction

**Figure 1:**
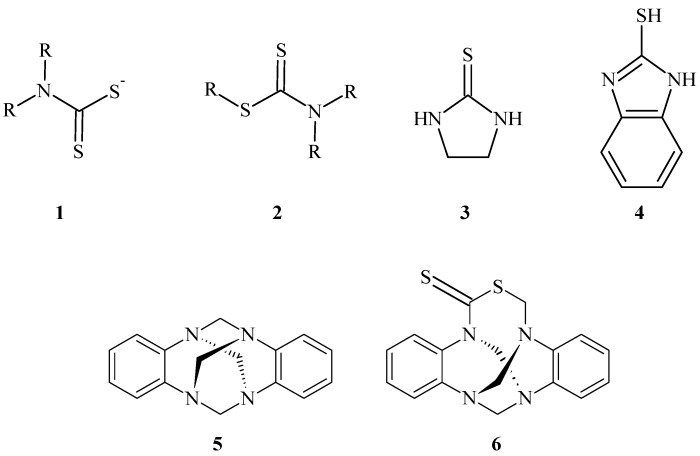
The chemical structures of compounds **1**–**6**.

The reaction between carbon disulfide and amines was originally reported by Hoffmann in the 19th century [1]. This reaction with primary and secondary aliphatic amines in ethanol yields reactive dithiocarbamate salt intermediates **1** that can be converted into S-alkyl dithiocarbamates **2**, which have several biological activities, via reaction with electrophiles or into dithiocarbamic acids by treatment with mineral acids [2]. In the case of aliphatic diamines, the treatment of the dithiocarbamate intermediate with acid gives cyclic thioureas **3** [3]. In contrast, the reaction with *o*-phenylenediamine under basic conditions afforded 2-mercaptobenzimidazole (**4**) [4,5] (Figure 1). In recent years, cyclic thioureas and mercaptobenzimidazoles have had important industrial applications as anticorrosive agents [6,7,8], friction attenuators [9] and heavy metal adsorbents [10]. Moreover, derivatives of cyclic thioureas have important roles in medicinal chemistry owing to their utility as antiseptic [11], antidepressive [12], antitumour [13], and antibacterial agents [14,15,16]. In addition, thioureas have been used as ligands in copper (I)-based complexes [17,18] and in complexes with other metals [19].

**Scheme 1:**
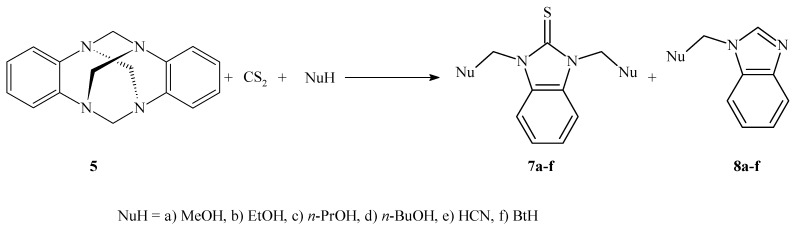
General reaction of **5** with nucleophiles in the presence of CS_2._

In continuation of our work with the benzoaminal 6*H*,13*H*-5:12,7:14-dimethanedibenzo[*d,i*]-[1,3,6,8]tetraazecine (DMDBTA, **5**) [20,21,22,23,24], we recently became interested in synthesizing the cyclic dithiocarbamate 8*H*,15*H*-5:14,9:16-dimethanedibenzo[*d,i*]-[1,3,6,8,11]thiatetraazatricycledo-decine-6-thione (**6**). To obtain this product, we planned to use DMDBTA (**5**) and carbon disulfide in ethanol, following the method reported by Donia *et al. * [25]. In earlier attempts, we found that the reaction of **5 **with carbon disulfide in ethanol did not provide the desired cyclic dithiocarbamate **6**. Instead, we found that **5** reacts with carbon disulfide to produce 1,3-bis(ethoxymethyl)-1,3-dihydro-2*H*-benzimidazole-2-thione (**7b**) and 1-(ethoxymethyl)-1*H*-benzimidazole (**8b**) (Scheme 1). 

The effects of the solvent on the product yields were examined using **5** as a substrate in various alcohols, including methanol, ethanol, *n*-propanol, *n*-butanol and *t*-butanol. Contrary to our expectations, when **5** was treated with *t*-butanol, the desired derivatives were not obtained. The efficacy of using other nucleophiles was demonstrated by reacting **5** with CS_2_ and either hydrogen cyanide or benzotriazole in the aprotic polar non-nucleophilic solvents 1,4-dioxane and acetonitrile, respectively.

## 2. Results and Discussion

We found the optimum conditions for the synthesis of compounds **7a–f** and **8a–f**. These products were obtained in good yields with high purities. Both the analytical and spectral data (IR, ^1^H-NMR, ^13^C-NMR and GC-MS analyses) of all synthesised 1,3-bis(alcoxymethyl)-1,3-dihydro-2*H*-benzimidazole-2-thiones **7a–f** and 1-(alkoxymethyl)-1,3-dihydro-2*H*-benzimidazole compounds **8a–f** were in full agreement with the proposed structures.

The IR data for benzimidazole-2-thiones **7a–d** clearly indicate the presence of the C=S group, with υ(C=S) vibrations at 1098, 1099, 1104 and 1108. In the 1H-NMR spectra, the N–CH_2_–O protons of the hemiaminal moiety of 1,3-bis(alcoxymethyl)-1,3-dihydro-2*H*-benzimidazole-2-thione appeared as a singlet at approximately 5.79–5.82 ppm. In the ^13^C-NMR spectra, the signal of the thiourea functional group was clearly observed at 171.8–169.3 ppm, and these signals are consistent with the signals of analogous molecules [26]. The attributions of the other signals in ^13^C-NMR spectra were based on the analysis of the HMQC and HMBC spectra. The molecular ions and fragment ions in the mass spectra were also consistent with the assigned structures. The reaction with other nucleophiles in the presence of an inert solvent under the same conditions produces benzimidazole and benzimidazole-2-thione derivatives. When the reaction was carried out with the cyanide anion, 1,3-bis(cyano-1-ylmethyl)-1,3-dihydro-2*H*-benzimidazole-2-thione (**7e**) and 1*H*-benzimidazol-1-yl-acetonitrile (**8e**) were obtained; in an analogous manner, the reaction with benzotriazole (BtH) afforded 1,3-bis(1*H*-1,2,3-benzotriazol-1-ylmethyl)-1,3-dihydro-2*H*-benzimidazole-2-thione (**7f**) and 1-(1*H*-benzimidazol-1-ylmethyl)-1*H*-1,2,3-benzotriazole (**8f**). The structures of these heterocyclic systems were confirmed by the spectroscopic data.

As shown in Table 1, the results reveal that the yield of the reaction depends on the size of the nucleophile. Consequently, the use of *t*-butanol under the same reaction conditions afforded complex mixtures, from which we were unable to isolate the expected benzimidazole-2-thione and 1-*H*-benzimidazole derivatives. 

**Table 1:** Yields of benzimidazole-2-thione (**7a–f**) and 1-*H*-benzimidazole (**8a–f**) derivatives.

Entry	NuH	7 (%)	8 (%)	t (h)
**a**	MeOH	48.5	51.4	24
**b**	EtOH	30.1	32.3	30
**c**	*n*-PrOH	23.3	24.5	34
**d**	*n*-BuOH	15.0	16.7	40
**e**	HCN	25.3	26.0	24
**f**	BtH	35.2	36.7	24

**Scheme 2:**
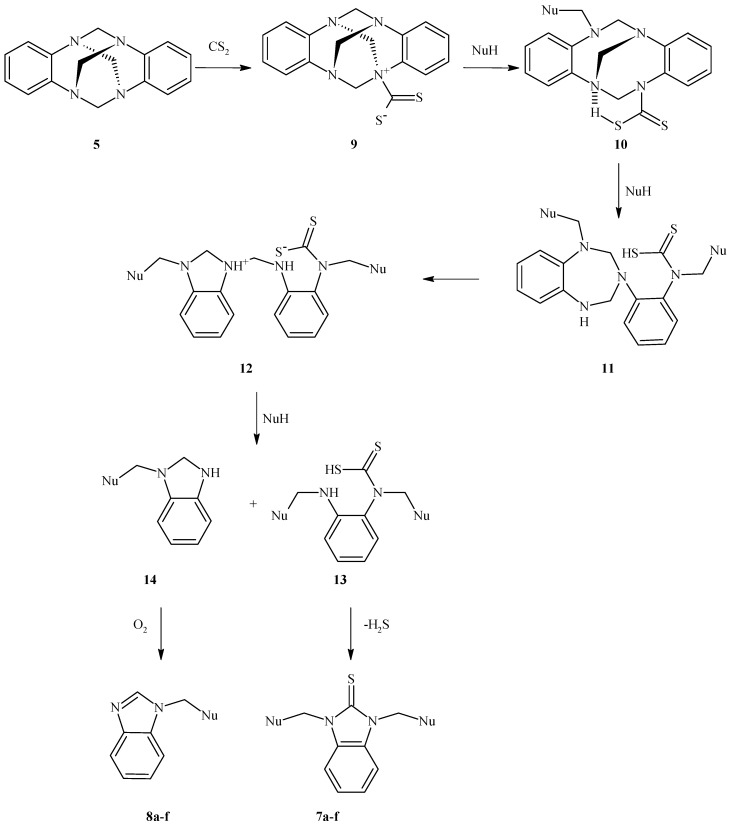
Proposed pathway for the formation of **7a–f** and **8a–f**.

According to these results, in the presence of an adequate nucleophile, the reactions efficiently proceed to provide the ring opening of the benzoaminal. Thus, the first step of the reaction between **5** and CS_2_ might be fast, and the second step, involving the nucleophilic attack of the dithiocarbamate salt, is slower and consequently should be the rate-limiting step. The onset of the reaction is indicated by the evolution of hydrogen sulphide, whose odour was noticeable during the reaction. Based on these results, we propose a possible pathway for the formation of the products (Scheme 2).

We assumed that carbon disulfide first reacts with **5** to form the expected dithiocarbamate salt **9** as a very active intermediate. This intermediate then reacts with one equivalent of the nucleophile, a reaction that includes a proton shift, to produce a second intermediate **10** that is able to form S–H···N intramolecular hydrogen bonds. This intramolecular interaction in this intermediate decreases the relative stability of the intermediate and induces the attack of a second equivalent of nucleophile to give a 1-substituted-3-aryl-2,3,4,5-tetrahydro-1*H*-1,3,5-benzotriazepine intermediate **11**, in which a proton transfer induces an intramolecular rearrangement to give **12**. The presence of a positive charge on the 1*H*-benzimidazole ring of **12** makes this adduct fairly labile, and the central *N*CH_2_*N* moiety in **12** undergoes a regioselective cleavage involving the preferential attack by a third equivalent of nucleophile to give **13** and **14**. Then, the benzimidazole-2-thiones**7a–f** are obtained by cyclisation of the acyclic intermediate **13** with the elimination of a molecule of H_2_S. Monosubstituted-benzimidazolines **14a–f**, the other products formed from **12** under these reaction conditions, smoothly undergo oxidation in air to yield the 1-(alkoxymethyl)-1,3-dihydro-2*H*-benzimidazole derivatives **8a–f**, as observed previously for other benzimidazoline derivatives [27]. Alcohols and cyclic ethers are good solvents for this oxidative process [28,29,30].

## 3. Experimental

### 3.1. General

Melting points were determined on an Electrothermal 9100 melting point apparatus and are uncorrected. Chemicals were used without further purification. FT-IR spectra were recorded in potassium bromide pellets using Thermo Nicolet IS10 spectrophotometer. ^1^H-NMR and ^13^C-NMR spectra were recorded in CDCl_3_ using a Bruker Avance AV-400 MHz spectrometer operates at 400 MHz for ^1^H and 100 MHz for ^13^C. Elemental analyses (C, H, N) were determined in a Thermo Scientific Flash 2000. Combined GC–MS analysis was performed on a Hewlett–Packard 5973 mass spectrometer at 70 eV coupled to a Hewlett–Packard 6890 gas chromatograph. 

*General procedure for the reaction of DMDBTA with CS_2_ in alcohols*: Following the general procedure described in the literature [25], carbon disulfide (0.95 mmol, 0.07 mL) was added dropwise over 30 min to a shaking solution of DMDBTA (0.95 mmol) in the desired alcohol (30 mL). This yellow solution was stirred at room temperature in the dark until the DMDBTA had dissolved completely. The reaction was monitored by TLC. The removal of the solvent at reduced pressure (50 mmHg) resulted in the collection of a resinous solid that was then purified via column chromatography on silica gel (elution using benzene:ethyl acetate in a 9:1 mixture).

*Procedure for the reaction of DMDBTA with benzotriazole and CS_2_*: A mixture of DMDBTA (0.95 mmol, 0.25 g), 1*H*-benzotriazole (2.85 mmol, 0.34 g) and carbon disulfide (0.95 mmol, 0.07 mL) was stirred in 1,4-dioxane (30 mL) for 24 h. at room temperature in the dark yielding a resinous solid. The precipitated solid was collected and purified via column chromatography on silica gel (elution using benzene: ethyl acetate in a 9:1 mixture).

*Procedure for the reaction of DMDBTA with cyanide anion and CS_2_*: To a solution of DMDBTA (0.95 mmol, 0.25 g) and carbon disulfide (0.95 mmol, 0.07 mL) in acetonitrile (15 mL), an excess of hydrogen cyanide was bubbled in slowly. The reaction mixture was stirred at room temperature in the dark for 24 h. The removal of the solvent at reduced pressure (50 mmHg) resulted in the collection of a resinous product that was then purified via column chromatography on silica gel (elution using benzene:ethyl acetate in a 9:1 mixture).

### 3.2. Physical and Spectral Data

*1,3-Bis(methoxymethyl)-1,3-dihydro-2H-benzimidazole-2-thione* (**7a**): White solid; m.p. 99–101 °C; ^1^H-NMR d: 3.24 (6H, s, –CH_3_), 5.79 (4H, s, N-CH_2_-O), 7.24 (2H, m), 7.35 (2H, m); ^13^C-NMR d: 52.6, 74.3, 111.5, 121.9, 132.5, 171.8; IR ν_max_ (cm^–1^): 1,108 (C=S), 1,285 (C-O); EIMS, 70 eV, *m/z*: 238 (M^+^); Anal. Calcd. for C_11_H_14_N_2_O_2_S (238.36): C, 55.44; H, 5.92; N, 11.76; S, 13.46. Found: C, 55.23; H, 5.96; N, 11.46; S, 13.22.

1,3-Bis(ethoxymethyl)-1,3-dihydro-2H-benzimidazole-2-thione (**7b**): Melting point 103–104 °C (lit. [31] 104–106 °C).

*1,3-Bis(propoxymethyl)-1,3-dihydro-2H-benzimidazole-2-thione* (**7c**): White solid; m.p. 120–122 °C; ^1^H-NMR d: 0.91 (6H, t, *J* = 6.9 Hz, –CH_3_), 1.59 (4H, s, *J* = 6.9 Hz, O-CH_2_CH_2_CH_3_), 3.46 (4H, q, *J* = 6.9 Hz, O-CH_2_CH_2_CH_3_), 5.82 (4H, s, N–CH_2_–O), 7.27 (2H, m), 7.39 (2H, m); ^13^C-NMR d: 10.7, 22.5, 64.9, 73.7, 110.3, 123.8, 132.4, 169.3; IR ν_max_ (cm^–1^): 1,098 (C=S), 1,289 (C-O); EIMS, 70 eV, *m/z*: 294 (M^+^); Anal. Calcd. for C_15_H_22_N_2_O_2_S (294.40): C, 61.19; H, 7.53; N, 9.52; S, 10.89. Found: C, 61.05; H, 7.66; N, 9.48; S, 10.85.

*1,3-Bis(butoxymethyl)-1,3-dihydro-2H-benzimidazole-2-thione * (**7d**): White solid; m.p. 124–126 °C; ^1^H-NMR d: 0.84 (6H, t, *J* = 6.7 Hz, –CH_3_), 1.65–1.68 (8H, m, O-CH_2_CH_2_CH_2_CH_3_), 3.59 (4H, t, *J* = 6.7 Hz, O-CH_2_CH_2_ CH_2_CH_3_), 5.82 (4H, s, N-CH_2_-O), 7.30 (2H, m), 7.43 (2H, m); ^13^C-NMR d: 14.3, 18.3, 30.9, 67.4, 72.4, 110.3, 123.6, 131.8, 171.1; IR ν_max_ (cm^–1^): 1099 (C=S), 1288 (C-O); EIMS, 70 eV, *m/z*: 322 (M^+^); Anal. Calcd. for C_17_H_26_N_2_O_2_S (322.46): C, 63.32; H, 8.13; N, 8.69; S, 9.94. Found: C, 63.24; H, 8.16; N, 8.67; S, 9.91.

*1,3-bis(Cyano-1-methyl)-1,3-dihydro-2H-benzimidazole-2-thione* (**7e**): Oily product; ^1^H-NMR d: 5.32 (4H, s, N-CH_2_-CN), 7.56 (2H, m), 7.73 (2H, m); ^13^C-NMR d: 31.9, 111.9, 118.3, 119.4, 134.7, 179.4; IR ν_max_ (cm^–1^): 2,236 (CN) 1,102 (C=S), 1,285; EIMS, 70 eV, *m/z*: 228 (M^+^); Anal. Calcd. for C_11_H_8_N_4_S (228.27): C, 57.88; H, 3.53; N, 24.54; S, 14.05. Found: C, 57.91; H, 3.56; N, 24.48; S, 13.91.

*1,3-bis(1H-1,2,3-Benzotriazol-1-ylmethyl)-1,3-dihydro-2H-benzimidazole-2-thione* (**7f**): Yellow Solid; m.p. 142–144 °C; ^1^H-NMR d: 7.07 (4H, s, N-CH_2_-Bt), 7.21 (4H, m, benzimidazoline nucleus), 7.35 (2H, t, *J* = 7.8 Hz, benzotriazole nucleus), 7.52 (2H, t, *J* = 7.8 Hz, benzotriazole nucleus), 8.08 (2H, d, *J* = 7.8 Hz, benzotriazole nucleus), 8.43 (2H, d, *J* = 7.8 Hz, benzotriazole nucleus); ^13^C-NMR d: 55.3, 110.1, 111.7, 119.9, 124.0, 124.6, 128.5, 130.2, 132.6, 146.2, 169.6. IR ν_max_ (cm^−1^): 1108 (C=S), 1,271 (C=N); EIMS, 70 eV, m/z: 412 (M^+^); Anal. Calcd. for C_21_H_16_N_8_S (412.45): C, 61.15; H, 3.91; N, 27.17; S, 7.77. Found: C, 61.23; H, 3.96; N, 27.26; S, 7.55.

## 4. Conclusions

In conclusion, the reported synthesis is reasonably efficient, direct, and operationally simple. We believe that the methodology presented herein can have wide applications for the development of synthetically useful benzimidazole-2-thiones that were previously inaccessible by other routes.
